# The relaxation effect of autonomous sensory meridian response depends on personal preference

**DOI:** 10.3389/fnhum.2023.1249176

**Published:** 2023-12-05

**Authors:** Noriko Sakurai, Kazuaki Nagasaka, Kei Sasaki, Yukina Yuguchi, Shingo Takahashi, Satoshi Kasai, Hideaki Onishi, Naoki Kodama

**Affiliations:** ^1^Department of Radiological Technology, Niigata University of Health and Welfare, Niigata, Japan; ^2^Department of Physical Therapy, Niigata University of Health and Welfare, Niigata, Japan; ^3^Graduate School of Health and Welfare, Niigata University of Health and Welfare, Niigata, Japan; ^4^Department of Healthcare Informatics, Takasaki University of Health and Welfare, Gunma, Japan

**Keywords:** ASMR, insular cortices, amygdala, relaxation, fMRI

## Abstract

**Background:**

Autonomous sensory meridian response (ASMR) is a sensory response such as tingling and pleasantness from audiovisual stimuli. ASMR videos come in a wide variety of types, and personal preferences are biased. There are many reports of the effects os ASMR on sleep onset, anxiety relief, and other relaxation effects. However, prior task-oriented studies have used ASMR videos provided by the experimenter. We hypothesized that ASMR movies of a personal preference would show significantly increased activity in the nucleus accumbens, frontal cortex, and insular cortex, which are brain areas associated with relaxation. Therefore, the purpose of this study was to elucidate the neuroscientific basis for the relaxation effects of ASMR videos that match someone’s personal preferences.

**Methods:**

This study included 30 healthy individuals aged ≥18 years. ASMR enthusiasts were included as the target population due to the need to have a clear preference for ASMR videos. A control video (1 type) and ASMR videos (20 types) were used as the stimulus tasks. Among the ASMR videos, those with high and low evaluation scores were considered liked and dislikedASMR videos, respectively. Functional magnetic resonance imaging was performed while the participants viewed a block design with a resting task in between. The data were analyzed using Statistical Parametric Mapping 12 to identify the areas activated by control, disliked, and liked ASMR videos.

**Results:**

Emotion-related areas (the amygdala, frontal cortex, and insular cortex) not activated by control and unliked ASMR videos were activated only by liked ASMR videos.

**Conclusion:**

The amygdala, frontal cortex, and insular cortex may be involved in the limbic dopamine circuits of the amygdala and middle frontal gyrus and the autonomic balance of the left and right insular cortices. This suggests the potential of positive mood and its use as a treatment for patients with anxiety and depression. These results suggest that the use of ASMR videos to match individual preferences may induce relaxation and have beneficial effects on depression and other disorders, and also support the introduction of ASMR videos in mental health care.

## 1 Introduction

Autonomous sensory meridian response (ASMR) is a pleasant sensory response triggered by audiovisual stimuli. The reaction, called tingling, begins at the scalp, spreads to the neck and shoulders, and then to the back and limbs (Barratt and Davis, 2015). ASMR videos that cause tingling can be viewed on YouTube and other platforms. The triggers that cause the tingling are very personal. The videos include whispers, soft speech, tapping and scratching sounds, slow and expert hand movements, and meticulous attention (Barratt and Davis, 2015; Poerio et al., 2018). These videos have gained a large online community of young people in recent years.

The reported uses of ASMR include sleep induction, relaxation, and anxiety relief (Barratt and Davis, 2015; Barratt et al., 2017). Many studies have reported the effects of ASMR. In a physiological study, Poerio et al. (2018) reported contradictory results, with increased skin conductance and decreased heart rate. Engelbregt et al. (2022) reported that ASMR videos induced relaxation in individuals insensitive to ASMR, with evidence of a reduced heart rate. Valtakari et al. (2019) observed increased pupil diameter during tingling. Electroencephalography (EEG) studies have reported the sleep-inducing effects of ASMR and have proposed that this method can improve sleep quality (Lee et al., 2019; Vardhan et al., 2020). Fredborg et al. (2021) reported relaxation from increased α waves. Functional magnetic resonance imaging (fMRI) studies have also demonstrated brain function during tingling in areas involved in social behavior, reward systems, and empathy. The studies also suggested the contribution of oxytocin release to the relaxed state during tingling (Lochte et al., 2018). Lee et al. (2020) observed increased functional connectivity in the medial prefrontal cortex during the resting state and ASMR video viewing. This suggests the involvement of mentalization and self-referential processing in its induction and maintenance. We also compared the relaxation effects of ASMR and classical music by fMRI. In both cases, activation of the thalamus and other areas involved in sleep was observed, particularly that of the medial prefrontal cortex, which is involved in inducing relaxation in ASMR (Sakurai et al., 2021). In addition, we compared the effects of ASMR video and sound alone. Participants who watched the video showed activation of the dopamine pathway in the limbic system, suggesting that a positive mood can reduce stress and improve depression and that sound alone can balance the autonomic nervous system by activating the left and right insular cortices (Sakurai et al., 2023). Our study showed that relaxation was induced regardless of tingling.

ASMR does not occur in everyone. Roberts et al. (2020) stating that 28% consider themselves to be ASMR responders, while Swart et al. (2021) reported that 38% consider themselves to be ASMR responders. Because tingling is a subjective aspect of an individual, an ASMR video that induces tingling in one individual may not induce it in another (Barratt et al., 2017) ASMR videos have been shown to produce relaxation and positive mood even when tingling does not occur (Barratt and Davis, 2015). Barratt and Davis (2015) classified ASMR into four categories (whispering, personal attention, crisp sounds, and slow movements), whereas Fredborg et al. (2017) classified these sounds into five categories (watching, touching, repetitive, simulations, and mouth sounds). Thus, many categories and composites of these categories exist. Moreover, many other ASMR videos do not fit into these categories, making the content broad. This finding supports the hypothesis that individual preferences are highly biased.

In a study focusing on personal differences, Smith et al. (2019) investigated individual differences in sensitivity to five ASMR categories using resting-state fMRI. Functional connectivity was negatively correlated with ASMR sensitivity but positively correlated in the dorsal attention network, suggesting that ASMR is an attention-driven process. A more recent study developed a trigger checklist for ASMR responders consisting of 37 triggers, focusing on personal differences (Poerio et al., 2023).

Thus, it is clear that ASMR is highly influenced by personal differences, but studies tailored to personal differences are lacking because of the wide variety of types. In many previous studies of task-oriented ASMR research, the ASMR videos for the task were prepared by the experimenter and used by the subject (Lochte et al., 2018; Lee et al., 2019, 2020). This study is the first to use ASMR videos tailored to individual preferences and to prove their effectiveness based on brain function mechanisms. In many previous studies, the ASMR videos for the task were prepared by the experimenter and used by the participant. This study is the first to use ASMR videos tailored to individual preferences and to prove their effectiveness based on functional brain mechanisms.

Brain regions linked to the relaxation effects of ASMR, as inferred from our previous studies, suggest the involvement of the nucleus accumbens, prefrontal cortex, and insular cortex (Sakurai et al., 2021, 2023). As such, we hypothesized that the activity in these brain areas would be significantly increased in response to the preferred ASMR videos compared to that of ASMR videos of no particular interest. Therefore, the purpose of this study was to elucidate the neuroscientific basis for the relaxation effects of ASMR videos that match individual preferences because ASMR videos are personal and subjective.

## 2 Materials and methods

### 2.1 Participants

This study included 30 healthy participants (20 men and 10 women, mean age 20.1 ± 1.3 years) aged ≥18 years. The target population consisted of only ASMR enthusiasts because of the need for a clear preference for certain ASMR categories. However, the participants were required not to watch ASMR for 1 week before the experiment. This study was approved by the Research Ethics Committee of Niigata University of Health and Welfare (approval no. 18961-221208). Written informed consent was obtained from all participants. In addition, a medical interview was conducted to ensure the safety of the MRI.

### 2.2 Stimulus tasks

The stimulus tasks involved viewing a control video (1 type) and ASMR videos (20 types). For the control task, we prepared an “educational/demonstration video.” This video did not include gentle speech or slow, delicate hand movements, as employed in the ASMR videos (Poerio et al., 2018). ASMR videos were prepared to cover all five categories (Fredborg et al., 2017): watching, touching, repetitive sounds, simulations, and mouth sounds. There were five types of watching videos (turning pages, writing sounds, speaking words slowly, pouring soda, shaving soap), two kinds of touching videos (touching hand motions, ear-pulling), six kinds of repetitive sounds videos (holding slime, typing, rolling marbles, tapping nails, cutting vegetables, grilling meat), four types of simulation videos (shampoo, haircut, teeth brushing lecture, calm massage), and three kinds of mouth sounds videos (eating fried fish, eating salmon, eating noodles). Of these 20 ASMR videos, the videos that were liked and disliked were determined. The participants watched all 20 types of ASMR at least 1 week before the experiment and scored each for two mood types: relaxed mood and tingling mood (Sakurai et al., 2023). For each of the 20 ASMR videos, the degree to which they evoked more emotion than the control video was rated on a visual analog scale from 0 to 10. The two mood scores were recorded, and the videos with the highest and lowest total scores were defined as the favorite and least favorite ASMR videos, respectively. In the case of a tie, participants were asked to verbally indicate which video they preferred or did not like better. The resting task was presented with a fixed cross on a black screen while the subject listened to white noise. We used white noise as an emotionless and random signal with equal power at any frequency in a specified bandwidth (Cardona et al., 2020).

### 2.3 Block design

**FIGURE 1:**
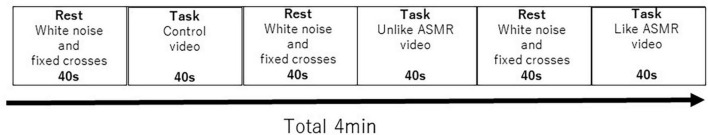
Block design. The order of control, disliked ASMR, and liked ASMR videos in the task was random. ASMR, autonomous sensory meridian response.

The block design consisted of alternating repetitions of 40 s for the rest period and 40 s for the stimulus task for a total of 4 min. The order in which the stimulus tasks were presented was random. White noise and fixed crosses interspersed between stimulation tasks returned the cerebral blood oxygen signal to baseline and separated the stimulation times. Video editing was performed using PowerDirector 18 (CyberLink, Taipei, Taiwan) to create compressed audio data in MP4 format with all volume settings set to 98 dB. The block design is illustrated in Figure 1.

### 2.4 Apparatus

Imaging was performed using a 3-T MRI system (Vantage Galan; Canon Medical Systems, Tochigi, Japan) with a 16-channel head coil. The participant lay in the MRI machine and watched the block design. The images were displayed on a magnetic resonance theater (Canon Medical Systems) and were viewed while the participant lay on his back. The sound source was an MRI headphone system (iMag; Star Products, Tokyo, Japan) with high sound insulation that blocks the scanning sounds.

### 2.5 MRI acquisition

High-resolution MRI scans were required to obtain detailed anatomical information before fMRI. For this purpose, a high-resolution T1-weighted magnetization-prepared rapid-gradient-echo (MP-RAGE) sequence was used to obtain structural images, with the following parameters: repetition time (TR) = 5.8 ms, echo time (TE) = 2.7 ms, inversion time (TI) = 900 ms, flip angle (FA) = 9, matrix = 256 × 256, field of view (FOV) = 230 × 230 mm, and slice thickness = 1.2 mm. Echoplanar imaging (EPI) was used to capture fMRI scans. The EPI scans were repeated, and images were captured to compare the two stimuli. The imaging conditions for fMRI to cover the entire brain were as follows: TR = 2,000 ms, TE = 25 ms, FA = 85°, matrix = 64 × 64, FOV = 240 × 240 mm, and slice thickness = 3 mm.

### 2.6 fMRI data analyses

The fMRI data were preprocessed and analyzed using Statistical Parametric Mapping 12 (Wellcome Trust Center for Neuroimaging, London, UK) in MATLAB (MathWorks, Natick, MA). After correcting the time difference using slice-timing correction, the displacement due to motion was corrected using realignment. Coagisters were applied to correct for misalignments between structural and functional images. The data were normalized by aligning each participant’s brain with the Montreal Neurological Institute standard brain coordinate template. The normalized images were smoothed using an 8-mm Gaussian kernel. After pretreatment, we used a general linear model (GLM) to identify changes in brain activity associated with liked and disliked ASMR videos and control videos by block design. Contrast images were created at the first level (single subject) using the following contrasts: (1) Liked ASMR = 1, Rest = 0; (2) Disliked ASMR = 1, Rest = 0; (3) Control = 1, Rest = 0; (4) Liked ASMR = 1, Disliked ASMR = −1. The contrast in (4) can be used to identify brain areas that show significantly increased activity in the non-preferred ASMR condition compared with that in the preferred ASMR condition. In the next group analysis (second level), a one-sample *t*-test was performed using the four aforementioned contrasts. The initial threshold for the voxel level was set at an uncorrected *p* < 0.001. Clusters were corrected for the false discovery rate (FDR), with those meeting *p* < 0.05 considered significant. Each of the three stimulus tasks was analyzed by subtracting disliked ASMR from liked ASMR. We also investigated the correlation between the relaxed and tingling mood scores for the liked and disliked ASMR videos.

## 3 Results

**FIGURE 2:**
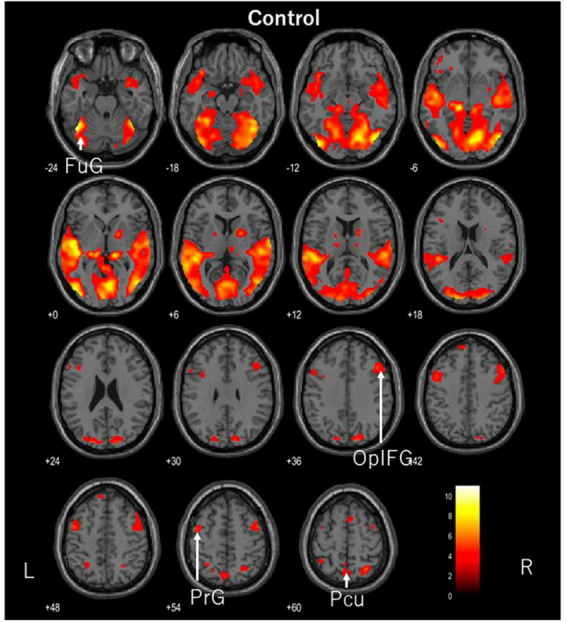
Axial images of the whole brain during viewing of the control video. The images show activation of the left fusiform gyrus (FuG), right opercular part of the inferior frontal gyrus (OplFG), left precentral gyrus (PrG), and left precuneus (Pcu).

**FIGURE 3:**
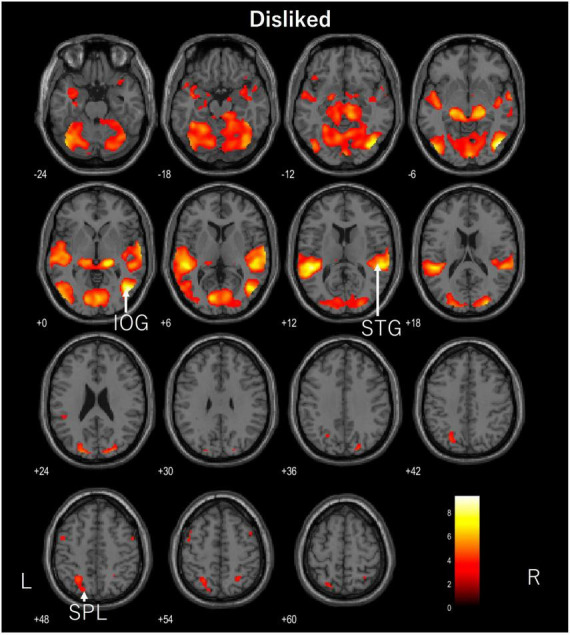
Axial images of the whole brain during viewing of the disliked ASMR video. The images show activation of the right inferior occipital gyrus (IOG), right superior temporal gyrus (STG), and left superior parietal lobule (SPL). ASMR, autonomous sensory meridian response.

**FIGURE 4:**
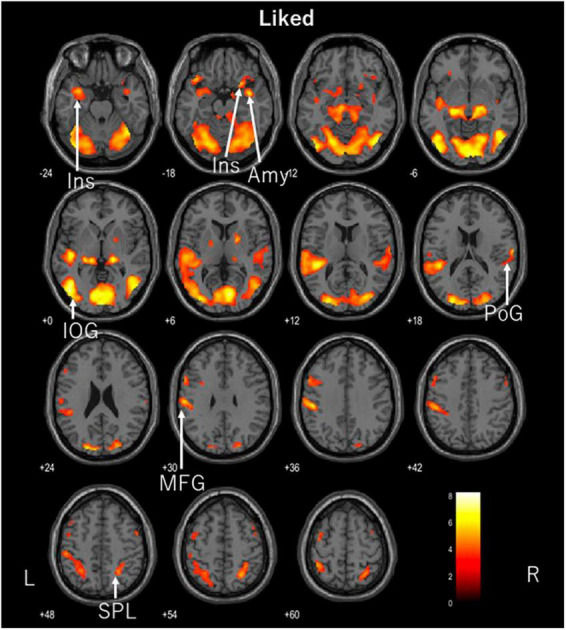
Axial images of the whole brain during viewing of liked ASMR videos. The images show activation of the left inferior occipital gyrus (IOG), right amygdala (Amy), right and left insula (Ins), right postcentral gyrus (PoG), right superior parietal lobule (SPL), and left middle frontal gyrus (MFG). ASMR, autonomous sensory meridian response.

The participants in this study were ASMR enthusiasts, none of whom reported feeling somatosensory experiences such as creepiness during the experiment. fMRI showed significant activation in the left fusiform gyrus, right opercular part of the inferior frontal gyrus, left precentral gyrus, and left precuneus when the control videos were being viewed (FDR corrected, *p* < 0.05). The disliked ASMR videos showed significant activation of the right inferior occipital gyrus, right superior temporal gyrus, and left superior parietal lobule (FDR corrected, *p* < 0.05). The liked ASMR videos showed significant activation in the left inferior occipital gyrus, right amygdala, right and left insula, right postcentral gyrus, right superior parietal lobule, and left middle frontal gyrus (FDR corrected, *p* < 0.05). Table 1 shows the statistics and coordinates of the areas that were significantly activated during the stimulation. The axial images of the areas activated during the stimulation task are shown in Figures 2–4. Analysis of liked ASMR videos subtracted from disliked ASMR videos revealed no significant activation sites. No sites showed any correlation with scores for the two moods.

**TABLE 1:** *P*-values and coordinates of the areas significantly activated during video viewing (control, disliked ASMR, and liked ASMR videos).

	Hemisphere	Locations	Cluster *p*-value (FDR)	Cluster size (voxels)	*T*-value	Z-score	x{mm}	y{mm}	z {mm}
Control	Left	Fusiform gyrus	<0.001	25333	11.05	6.87	−44	−58	−24
	Right	Opercular part of the inferior frontal gyrus	<0.001	722	5.42	4.47	44	24	34
	Left	Precentral gyrus	0.004	413	5.40	4.45	−48	0	52
	Left	Precuneus	0.004	387	5.13	4.29	−2	−54	66
Disliked	Right	Inferior occipital gyrus	<0.001	16787	9.40	6.32	48	−70	−4
	Right	Superior temporal gyrus	<0.001	2640	7.42	5.51	56	−32	12
	Left	Superior parietal lobule	0.004	378	4.71	4.03	−24	−58	50
Liked	Left	Inferior occipital gyrus	<0.001	16704	8.27	5.88	−44	−62	−2
	Right	Amygdala	0.044	190	5.89	4.74	38	2	−20
	Left	Insula	<0.001	578	5.57	4.55	−34	4	−22
	Right	Postcentral gyrus	<0.001	642	5.55	4.54	66	−18	20
	Right	Insula	0.034	218	5.46	4.49	26	14	−18
	Right	Superior parietal lobule	0.004	393	5.32	4.41	26	−58	52
	Left	Middle frontal gyrus	0.004	408	5.00	4.21	−56	12	32

## 4 Discussion

To our knowledge, this is the first study to identify the relaxation effects of ASMR videos based on personal preferences. We compared areas of the brain activated when viewing control, disliked, and liked ASMR videos. The temporal and occipital lobes were activated in the auditory and visual cortices, indicating their activation as large clusters because the task involved moving pictures. We also focused on other characteristic areas.

While the participants watched the control video, the left fusiform gyrus, right opercular part of the inferior frontal gyrus, left precentral gyrus, and left precuneus were activated. The fusiform gyrus is involved in the recognition of human facial expressions, while the opercular part of the inferior frontal gyrus serves as a functional link to various regions. The precentral gyrus is the primary motor cortex but is thought to be activated by eye movements. We believe that visual and somatosensory information is integrated within the precuneus. In this video, a man was lecturing in front of a whiteboard. Although he was expressionless, the person was facing the viewer and his entire face was visible. The lecturer’s facial expression and content may have been captured. Further study is needed on the selection of the control video.

The areas of activation in response to the unlinked ASMR videos were the inferior occipital gyrus, superior temporal gyrus, and superior parietal lobule. The inferior occipital gyrus is a part of the visual system that recognizes color and shape. The superior temporal gyrus is the primary auditory cortex, and the superior parietal lobule is involved in memory. Although the axial image appeared to show significant activation, it did not appear in any particular characteristic area and only in areas related to the visual and auditory cortices.

In contrast, the preferred ASMR videos showed activation in the visual and auditory areas of the inferior occipital gyrus, postcentral gyrus, and superior parietal lobule, as well as in the insular cortex, amygdala, and middle frontal gyrus. We previously compared the areas activated in the brain in response to ASMR videos and sound alone, in which the prefrontal cortex, including the nucleus accumbens, was activated by moving images, whereas the left and right insular cortices were activated by sound-only listening, suggesting that this led to relaxation (Sakurai et al., 2023). The activation of the frontal and insular cortices in this study was consistent with the hypothesis. No activation of the nucleus accumbens was observed; however, the amygdala was activated.

The amygdala plays a key role in emotional processing (Sergerie et al., 2008), is responsible for memory and learning emotional responses, and plays a major role in protecting against potential fear and danger (Adolphs, 1999). It is known primarily for its negative emotional response. We expected a correlation with the current tingling mood score, but there was no correlation. The amygdala, like the nucleus accumbens, is part of the network through which information circulates in the mesolimbic dopamine circuitry. Pleasurable emotions project dopamine to the nucleus accumbens via the frontal cortex, whereas the amygdala facilitates reward-seeking behavioral responses (Ambroggi et al., 2008). The amygdala is also an important part of the mesolimbic dopamine pathway, which links emotions to physiological responses. The amygdala was activated only on the right side. Regarding differences between the left and right brain hemispheres, the left hemisphere is significant for semantic material, such as language, while the right hemisphere is significant for non-semantic information, such as emotion (Markowitsch, 1998; Anderson and Phelps, 2001). In response to liked ASMR videos, the right amygdala, which may be involved in emotion processing, was activated. The mesolimbic dopamine circuit is also involved in stress, and is reported to be activated by social defeat stress and weakened by repeated social defeat stress. Furthermore, social defeat stress is a cause of psychiatric disorders such as depression (Tanaka et al., 2012).

Regarding the left-right difference in the insular cortices, the left and right insular cortices are involved in parasympathetic and sympathetic responses, respectively (Oppenheimer et al., 1992). In the present study, both the left and right insular cortices were activated. This state of autonomic balance is considered an interaction. This suggests that using liked ASMR videos may be more effective in inducing relaxation and autonomic balance than using control or disliked ASMR videos. The insular cortex also integrates the internal senses, which recognize the conscious perception of bodily responses, such as visceral sensation and autonomic control, with external senses, such as emotion and empathy (Uddin et al., 2017). This cross-sensory (decreased heart rate) and extrasensory (increased activity in somatosensory, motor, visual, and auditory cortices) activity is associated with the positive effects of ASMR on emotion via the autonomic nervous system (Poerio et al., 2022). We believe that the present study provides support that this cross-activity response occurs because of preferred ASMR videos, although not tingling.

An fMRI study by Datko et al. (2022) reported increased insular cortex activation after mindfulness training in patients with anxiety and depression (Datko et al., 2022), consistent with the insular cortical activation in the present study. Therefore, we believe that the effects of ASMR videos are similar to the inner receptive sensory awareness of mindfulness. Previously, Fredborg et al. (2018) suggested a similar effect to a mindfulness-based treatment program using ASMR. The increased relaxation and positive mood of ASMR also suggests that it may have a particularly beneficial effect on individuals with anxiety and depression (Barratt et al., 2017; Poerio et al., 2018; Smejka and Wiggs, 2022). This finding was further supported by functional brain mechanisms in the present study.

We hypothesized that ASMR videos would significantly increase activity in the nucleus accumbens, frontal cortex, and insular cortex, which are brain areas linked to the relaxation effect. The results showed activation in the amygdala, frontal cortex, and insular cortex in liked ASMR, suggesting involvement in mesolimbic dopamine circuits and autonomic balance. We propose that ASMR videos may be effective in counteracting depression and anxiety beyond relaxation by finding and selecting the ones you prefer. The results also support this from the viewpoint of brain function mechanisms, as viewing ASMR videos has already been incorporated into anxiety and depression prevention measures. We believe that this evidence provides further support for the effectiveness of ASMR.

However, this study has several limitations. First, comparisons with mindfulness were not performed. Thus, future comparative studies of the similarities and differences in mindfulness are needed. Second, the study did not evaluate participants with anxiety or depression; therefore, the effects are speculative. Finally, the connectivity in the activated areas should be tested using fMRI in real-life settings.

## 5 Conclusion

In the present study, emotion-related activation of the amygdala, frontal cortex, and insular cortex was observed only when participants watched their preferred ASMR videos. These areas may be involved in relaxation, autonomic balance, and mindfulness. The results support the introduction of ASMR videos into mental health care in the future, since ASMR videos are easy to access and can be used by anyone. It is desirable for more people to be aware of and use ASMR videos.

## Data availability statement

The original contributions presented in this study are included in this article/supplementary material, further inquiries can be directed to the corresponding author.

## Ethics statement

The studies involving humans were approved by the Research Ethics Committee of Niigata University of Health and Welfare. The studies were conducted in accordance with the local legislation and institutional requirements. The participants provided their written informed consent to participate in this study.

## Author contributions

NS and NK conceived the study and designed experiments. NS, KN, KS, YY, ST, and NK collected the MR data and performed the statistical analyses. NS and KN interpreted the data. NS, SK, HO, and NK drafted the manuscript. All authors approved the final version of the manuscript.

## References

[B1] Adolphs R. (1999). The human amygdala and emotion. *Neuroscientist* 5 125–137. 10.1177/107385849900500216

[B2] Ambroggi F. Ishikawa A. Fields H. L. Nicola S. M. (2008). Basolateral amygdala neurons facilitate reward-seeking behavior by exciting nucleus accumbens neurons. *Neuron* 59 648–661. 10.1016/j.neuron.2008.07.004 18760700 PMC2603341

[B3] Anderson A. K. Phelps E. A. (2001). Lesions of the human amygdala impair enhanced perception of emotionally salient events. *Nature* 411 305–309. 10.1038/35077083 11357132

[B4] Barratt E. L. Davis N. J. (2015). Autonomous sensory meridian response (ASMR): A flow-like mental state. *PeerJ* 3:e851. 10.7717/peerj.851 25834771 PMC4380153

[B5] Barratt E. L. Spence C. Davis N. J. (2017). Sensory determinants of the autonomous sensory meridian response (ASMR): Understanding the triggers. *PeerJ* 5:e3846. 10.7717/peerj.3846 29018601 PMC5633022

[B6] Cardona G. Rodriguez-Fornells A. Nye H. Rif-Ros X. Ferreri L. (2020). The impact of musical pleasure and musical hedonia on verbal episodic memory. *Sci. Rep.* 10:16113. 10.1038/s41598-020-72772-3 32999309 PMC7527554

[B7] Datko M. Lutz J. Gawande R. Comeau A. To M. N. Desel T. (2022). Increased insula response to interoceptive attention following mindfulness training is associated with increased body trusting among patients with depression. *Psychiatry Res. Neuroimaging* 327:111559. 10.1016/j.pscychresns.2022.111559 36308976 PMC12981234

[B8] Engelbregt H. J. Brinkman K. van Geest C. C. E. Irrmischer M. Deijen J. B. (2022). The effects of autonomous sensory meridian response (ASMR) on mood, attention, heart rate, skin conductance and EEG in healthy young adults. *Exp. Brain Res.* 240 1727–1742. 10.1007/s00221-022-06377-9 35511270 PMC9142458

[B9] Fredborg B. K. Champagne-Jorgensen K. Desroches A. S. Smith S. D. (2021). An electroencephalographic examination of the autonomous sensory meridian response (ASMR). *Conscious. Cogn.* 87:103053. 10.1016/j.concog.2020.103053 33232904

[B10] Fredborg B. K. Clark J. M. Smith S. D. (2018). Mindfulness and autonomous sensory meridian response (ASMR). *PeerJ* 6:e5414. 10.7717/peerj.5414 30123716 PMC6086079

[B11] Fredborg B. Clark J. Smith S. D. (2017). An examination of personality traits associated with autonomous sensory meridian response (ASMR). *Front. Psychol.* 8:247. 10.3389/fpsyg.2017.00247 28280478 PMC5322228

[B12] Lee M. Song C.-B. Shin G.-H. Lee S.-W. (2019). Possible effect of binaural beat combined with autonomous sensory meridian response for inducing sleep. *Front. Hum. Neurosci.* 13:425. 10.3389/fnhum.2019.00425 31849629 PMC6900908

[B13] Lee S. Kim J. Tak S. (2020). Effects of autonomous sensory meridian response on the functional connectivity as measured by functional magnetic resonance imaging. *Front. Behav. Neurosci.* 14:154. 10.3389/fnbeh.2020.00154 33192358 PMC7481390

[B14] Lochte B. C. Guillory S. A. Richard C. A. H. Kelley W. M. (2018). An fMRI investigation of the neural correlates underlying the autonomous sensory meridian response (ASMR). *Bioimpacts* 8 295–304. 30397584 10.15171/bi.2018.32 PMC6209833

[B15] Markowitsch H. J. (1998). Differential contribution of right and left amygdala to affective information processing. *Behav. Neurol.* 11 233–244. 10.1155/1999/180434 11568425

[B16] Oppenheimer S. M. Gelb A. Girvin J. P. Hachinski V. C. (1992). Cardiovascular effects of human insular cortex stimulation. *Neurology* 42 1727–1732. 10.1212/wnl.42.9.1727 1513461

[B17] Poerio G. L. Blakey E. Hostler T. J. Veltri T. (2018). More than a feeling: Autonomous sensory meridian response (ASMR) is characterized by reliable changes in affect and physiology. *PLoS One* 13:e0196645. 10.1371/journal.pone.0196645 29924796 PMC6010208

[B18] Poerio G. L. Ueda M. Kondo H. M. (2022). Similar but different: High prevalence of synesthesia in autonomous sensory meridian response (ASMR). *Front. Psychol.* 13:990565. 10.3389/fpsyg.2022.990565 36248469 PMC9558233

[B19] Poerio G. L. Osman F. Todd J. Kaur J. Jones L. Cardini F. (2023). From the outside in: ASMR is characterised by reduced interoceptive accuracy but higher sensation seeking. *Multisens. Res*. 10.1163/22134808-bja10108 37758236

[B20] Roberts N. Beath A. Boag S. (2020). A mixed-methods examination of autonomous sensory meridian response: Comparison to frisson. *Conscious. Cogn.* 86:103046. 10.1016/j.concog.2020.103046 33242764

[B21] Sakurai N. Nagasaka K. Takahashi S. Kasai S. Onishi H. Kodama N. (2023). Brain function effects of autonomous sensory meridian response (ASMR) video viewing. *Front. Neurosci.* 17:1025745. 10.3389/fnins.2023.1025745 36777643 PMC9909086

[B22] Sakurai N. Ohno K. Kasai S. Nagasaka K. Onishi H. Kodama N. (2021). Induction of relaxation by autonomous sensory meridian response. *Front. Behav. Neurosci.* 15:761621. 10.3389/fnbeh.2021.761621 34916914 PMC8669134

[B23] Sergerie K. Chochol C. Armony J. L. (2008). The role of the amygdala in emotional processing: A quantitative meta-analysis of functional neuroimaging studies. *Neurosci. Biobehav. Rev.* 32 811–830. 10.1016/j.neubiorev.2007.12.002 18316124

[B24] Smejka T. Wiggs L. (2022). The effects of autonomous sensory meridian response (ASMR) videos on arousal and mood in adults with and without depression and insomnia. *J. Affect. Disord.* 301 60–67. 10.1016/j.jad.2021.12.015 34915083

[B25] Smith S. D. Fredborg B. K. Kornelsen J. (2019). Atypical functional connectivity associated with autonomous sensory meridian response: An examination of five resting-state networks. *Brain Connect.* 9 508–518. 10.1089/brain.2018.0618 30931592 PMC6648236

[B26] Swart T. R. Bowling N. C. Banissy M. J. (2021). ASMR-Experience Questionnaire (AEQ): A data-driven step towards accurately classifying ASMR responders. *Br. J. Psychol.* 113 68–83. 10.1111/bjop.12516 34120330

[B27] Tanaka K. Furuyashiki T. Kitaoka S. Senzai Y. Imoto Y. Segi-Nishida S. E. (2012). Prostaglandin E2-mediated attenuation of mesocortical dopaminergic pathway is critical for susceptibilitly to repeated social defeat stress in mice. *J. Neurosci.* 32 4319–4329. 10.1523/JNEUROSCI.5952-11.2012 22442093 PMC3784244

[B28] Uddin L. Q. Nomi J. S. Hébert-Seropian B. Ghaziri J. Boucher O. (2017). Structure and function of the human insula. *J. Clin. Neurophysiol.* 34 300–306. 10.1097/WNP.0000000000000377 28644199 PMC6032992

[B29] Valtakari N. V. Hooge I. T. C. Benjamins J. S. Keizer A. (2019). An eye-tracking approach to autonomous sensory meridian response (ASMR): The physiology and nature of tingles in relation to the pupil. *PLoS One* 14:e0226692. 10.1371/journal.pone.0226692 31877152 PMC6932793

[B30] Vardhan V. V. Venkatesh U. Yadav S. (2020). “Signal processing based autonomous sensory meridian response to treat insomnia,” in *Proceedings of the International Conference on Electronics and Sustainable Communication Systems (ICESC). Coimbatore, India, 2-4 July 2020*, Coimbatore. 10.1109/ICESC48915.2020.9155950

